# Evaluation of a Compact Hybrid Brain-Computer Interface System

**DOI:** 10.1155/2017/6820482

**Published:** 2017-03-08

**Authors:** Jaeyoung Shin, Klaus-Robert Müller, Christoph H. Schmitz, Do-Won Kim, Han-Jeong Hwang

**Affiliations:** ^1^Machine Learning Group, Berlin Institute of Technology, Berlin, Germany; ^2^Department of Brain and Cognitive Engineering, Korea University, Seoul, Republic of Korea; ^3^NIRx Medizintechnik GmbH, Berlin, Germany; ^4^Department of Biomedical Engineering, Chonnam National University, Yeosu, Republic of Korea; ^5^Department of Medical IT Convergence Engineering, Kumoh National Institute of Technology, Gumi, Republic of Korea

## Abstract

We realized a compact hybrid brain-computer interface (BCI) system by integrating a portable near-infrared spectroscopy (NIRS) device with an economical electroencephalography (EEG) system. The NIRS array was located on the subjects' forehead, covering the prefrontal area. The EEG electrodes were distributed over the frontal, motor/temporal, and parietal areas. The experimental paradigm involved a Stroop word-picture matching test in combination with mental arithmetic (MA) and baseline (BL) tasks, in which the subjects were asked to perform either MA or BL in response to congruent or incongruent conditions, respectively. We compared the classification accuracies of each of the modalities (NIRS or EEG) with that of the hybrid system. We showed that the hybrid system outperforms the unimodal EEG and NIRS systems by 6.2% and 2.5%, respectively. Since the proposed hybrid system is based on portable platforms, it is not confined to a laboratory environment and has the potential to be used in real-life situations, such as in neurorehabilitation.

## 1. Introduction

Brain-computer interfaces (BCIs) assist people who cannot use their muscles to communicate with the external environment. One of the early uses of BCIs was to aid communication with people who have severe impairment of muscle movement, for instance, late-stage (“locked-in”) patients with amyotrophic lateral sclerosis (ALS, also known as Lou Gehrig's disease) [1]. Thanks to the rapid advance of neuroimaging modalities, BCI technology has broadened its application areas into the game industry, entertainment, and social neuroscience, for example, by providing alternative communication methods [2–6].

BCIs can be established by means of several brain imaging modalities, such as near-infrared spectroscopy (NIRS) [7], electroencephalography (EEG) [8], functional magnetic resonance imaging (fMRI) [9], magnetoencephalography (MEG) [10], and electrocorticogram (ECoG) [11]. Invasive BCI systems, such as ECoG-based BCIs, generally involve risks associated with the surgical operation for implanting microelectrodes in the brain and are thus limited for many potential BCI users. MEG- and fMRI-based systems only allow stationary and time-limited use due to their cost, complexity, size, and restricting environment. More compact and economical neuroimaging technologies, such as EEG or NIRS, hold the promise of providing lightweight, portable BCI systems for continuous use in more unrestrained and natural settings outside the lab, creating the opportunity for many new applications, such as neurorehabilitation.

EEG-based BCI systems have been most commonly used for rehabilitation training and for providing communication and control channels to individuals with limited motor functions [12–15]. A lightweight EEG-based BCI system with acceptable performance has been established but is often prone to drawbacks such as low signal-to-noise ratio (SNR) and susceptibility to motion artifacts and volume conduction [16–19].

NIRS is an emerging neuroimaging modality that records the cortical hemodynamic response based on changes in local optical transmission as measured by pairs of near-infrared light sources and detectors placed on the scalp surface [20]. This method is less sensitive to motion artifacts compared to EEG [21]. NIRS has by now been recognized as a promising neuroimaging modality that has overcome some of the drawbacks of EEG [22]. Recently, portable and cost-effective NIRS systems have become available [23] and have been actively used in the field of rehabilitation [24–26]. A defining characteristic of NIRS is the inherent delay of the measured hemodynamic response on the order of several seconds (typically > 5 s) [27], which limits its use in time-critical BCI applications and which requires a relatively long interstimulus interval (ISI) to gain task-relevant responses of reasonable quality. The resulting increase of the experimental time not only drops the overall information transfer rate usually quantified by bit rate per minute but can also exhaust the NIRS-based BCI users more easily [28].

To overcome the disadvantages of these individual methods, NIRS-EEG hybrid (HYB) BCI systems have been suggested to take advantage of superior performance provided by combining both modalities [29–36]. However, despite the comparatively low cost and compactness of both EEG and NIRS systems, the experimental setup of a hybrid system still poses practical challenges, even in a laboratory environment. Until now, each system required an individual amplifier, recording platform, and its own leads, which need to be affixed to the scalp with reliable optical and electrical contact. This poses added challenges and leads to generally increased setup times for HYB systems [32, 37, 38].

To date, to the best of our knowledge, no study that aims to reduce the complexity of hybrid NIRS-EEG BCI systems and validate their performance has been reported. In this study, we implement a lightweight and portable NIRS-EEG hybrid instrument and demonstrate its use for a hybrid BCI that has the potential for mobile and continuous use. We recorded NIRS and EEG signals simultaneously while the subject performed a word-picture matching test using simple mental arithmetic (MA), which is similar to the task in Power et al. [39]. The proposed hybrid system was validated by comparing its classification accuracies to those of the unimodal systems (EEG and NIRS).

## 2. Materials and Methods

### 2.1. Subjects

Eleven right-handed healthy subjects participated voluntarily in the experiment (1 male and 10 females, average age: 25.7 ± 3.2 years [mean ± standard deviation]). None of them had a history of neurological, psychiatric, or other disorders that might affect the experimental results. A written experiment summary was given to the participants, and each participant signed a written consent form prior to the experiment and obtained a financial reimbursement after the experiment. This study was approved by the Ethics Committee of the Institute of Psychology and Ergonomics, Berlin Institute of Technology (approval number: SH_01_20150330).

### 2.2. Apparatus

In the experiment, 14 EEG electrodes and eight NIRS probes (5 sources and 3 detectors) were placed on the scalp by means of a stretchy fabric cap (EASYCAP GmbH, Herrsching am Ammersee, Germany). The EEG system used was an EPOC device (Emotiv Inc., San Francisco, USA) and was selected for easy setup, wireless form factor, and, in particular, its economical price. The system had been verified in previous studies to show comparable performance to other commercial EEG devices with much higher prices [40–49].

**Figure 1:**
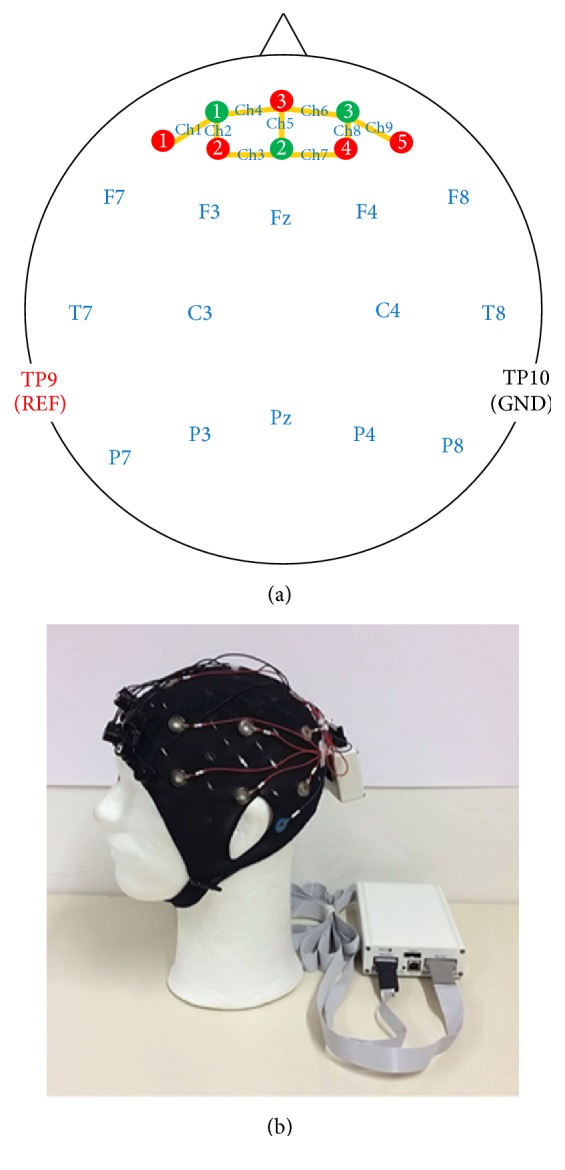
Channel layout of near-infrared spectroscopy (NIRS; Ch1–Ch9) and electroencephalography (EEG; (a)) and a headgear setup on a phantom head (b). Five sources (red circles, 1–5) and three detectors (green circles, 1–3) are located around Fp1, Fpz, and Fp2. Fourteen electrodes are located at Fz, F3, F4, F7, F8, C3, C4, T7, T8, Pz, P3, P4, P7, and P8. Reference and ground electrodes are located on TP9 and TP10, respectively.

In its original state, the EPOC uses a rigid headpiece of headphone-like appearance, which would not have allowed easy integration with NIRS. In a recent study, Debener et al. [50] demonstrated performance enhancement of the EPOC system by replacing the original head gear with a traditional fabric cap and ring electrodes. Following Debener et al.'s instructions, we dismantled the original hardware and moved the amplifier electronics into a small custom plastic case attached to the back of the cap. To provide good skin contact, we used passive Ag-AgCl ring electrodes (EASYCAP GmbH) with conductive gel. To measure task-related brain activation, a custom channel layout was chosen according to the international 10-10 system [51]. Fourteen electrodes were placed on frontal (F7, F3, Fz, F4, and F8), motor/temporal (C3 and C4/T7 and T8), and parietal (P7, P3, Pz, P4, and P8) areas. Reference and ground electrodes were attached on the left (TP9) and right (TP10) mastoids, respectively. The EEG signals were sampled at a 128 Hz sampling rate with provided software named “test bench” from the manufacturer. A portable NIRS system (NIRSport, NIRx Medical Technologies, NY, USA) was used to map hemodynamic responses. Five sources and three detectors were located over the prefrontal area around Fpz, Fp1, and Fp2 with an interoptode distance of 30 mm. Adjacent pairs of source and detector optodes comprised nine physical channels. NIRS signals were recorded at a 12.5 Hz sampling rate with NIRStar software, provided by the manufacturer.  Figure 1 shows the channel layout of NIRS optodes and EEG electrodes and the headgear setup on a phantom head.

### 2.3. Experimental Protocol

Subjects sat still in a comfortable armchair in front of a 24-inch LCD monitor. NIRS and EEG signals were acquired simultaneously from each subject while performing MA as a cognitive task and rest condition as a baseline task (BL). During MA, the subjects were instructed to subtract a single-digit number (between 6 and 9) from a random three-digit number and subtract it again from the result over and over as fast as possible until the trial ended (e.g., 544 − 7 = 537, 537 − 7 = 530, and 530 − 7 = 523). During the BL task, they were instructed not to think anything to maintain a low cognitive load state, while moving the body as little as possible. Even though the subject was instructed not to move the head and body, unintended subtle movement and unavoidable ocular movement might occur during the experiment. The quality of the EEG signal is easily affected by such artifacts, while the quality of the NIRS signal is less vulnerable to them.

**Figure 2:**
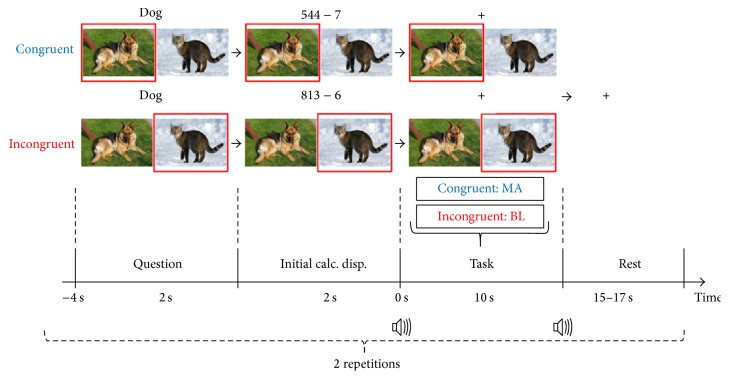
Timing sequence diagram of a single trial for the Stroop word-picture matching test. The whole process was done twice consecutively for congruent and incongruent tasks, which comprised a single trial. “Congruent first-incongruent later” and “incongruent first-congruent later” tasks were randomly presented. At the task presentation, the left- or right-side picture was sequentially selected. The name of either picture was displayed for 2 s. At initial mental arithmetic (MA) problem presentation, an example of a three-digit number minus a one-digit number (6 to 9) was shown instead of the name for 2 s. In a task period starting with a short beep (250 ms) and black fixation cross, subjects performed MA or baseline (BL) task if the word and picture were matched (congruent) or mismatched (incongruent), respectively. After 10 s, a rest period started with a short beep (250 ms), and a large black fixation cross was displayed at the center of the screen.

Figure 2 presents a timing sequence of a single trial. The experiment was designed as a Stroop word-picture test. A similar task was used in Power et al. [39]. A single trial was composed of task presentation (congruent or incongruent task, 2 s), followed by initial MA problem presentation (2 s), task period (10 s), and rest period (15–17 s). At the task presentation stage, two pictures (e.g., animals, fruits, and sport activities) were displayed on the screen side by side, and the name of either of the two objects was shown on the top of the screen. First, the left picture was highlighted using a red box. After 2 s, a random MA problem replaced the word. After 2 s, the problem was replaced by a black fixation cross with a short beep (250 ms) and the task period started. After the task period, a rest period with a random length of 15–17 s started with a short beep (250 ms), in which a large black fixation cross was displayed in the middle of screen. After the trial was finished, the same procedure was iterated with the right picture highlighted instead of the left one. If the displayed name matched the picture (congruent), subjects were asked to perform the MA task. On the other hand, if they were not matched (incongruent), subjects were asked to try not to think anything as a baseline task during the task period. During the rest period, subjects were instructed to relax and think nothing (BL). Therefore, congruent and incongruent trials were presented in a row as a pair, either “congruent first-incongruent later” or “incongruent first-congruent later,” for the same picture set. They were presented in a random order. A single trial consisted of both MA and BL trials, and a session consisted of 10 trials (i.e., 10 MA + 10 BL). After finishing a single session, a short break was given in which subjects were allowed to move their bodies but not to leave the seat. The session was repeated three times constituting three sessions. Overall, although the number of “congruent first-incongruent later” and “incongruent first-congruent later” trials might not be equal within each session, a total of 30 MA and 30 BL were acquired across the three sessions.

### 2.4. Data Analysis

#### 2.4.1. Point-Biserial Correlation Coefficient

A point-biserial correlation coefficient (*r-value*) is a measure of correlation between a dichotomous variable and a continuous variable. The* r-value* was estimated to determine the spectral and spatial distribution of separability. The* r-value* at the time of interest is defined as [52]

(1) $$\begin{array}{c} r\left(\;t\;\right)=\frac{\sqrt{N_{1}\cdot N_{2}}}{N_{1}+N_{2}}\frac{E\left[x\;|\;y=1\right]-E\left[x\;|\;y=2\right]}{\sigma \left[x\;|\;y\in \left\{1,2\right\}\right]}, \end{array}$$

where *t* ∈ [1,2,…, *T*]  and* t* is the length of the time of interest and *N*_1_ and *N*_2_ denote the total number of trials of class 1 and class 2 (MA and BL in this study), respectively. *x* denotes the data points that belong to class label *y* = 1 or 2. *E*[·] and *σ*[·] are mean and standard deviation operators, respectively. The *r*-value was also utilized to calculate the most discriminative frequency band for the EEG feature extraction and a spatial distribution of separability for the NIRS temporal response.

#### 2.4.2. Preprocessing

Offline EEG and NIRS data analyses were performed using MATLAB 2013b (The MathWorks, Natick, USA), in particular with the EEGLAB toolbox and BBCI toolbox [2, 53]. For NIRS data, raw light intensity signals were band-pass filtered (3rd-order Butterworth zero-phase filter with a passband of 0.01–0.2 Hz). Concentration changes of oxyhemoglobin (Δ[HbO]) and deoxyhemoglobin (Δ[HbR]) were then calculated according to the modified Lambert–Beer law [54, 55]. Baseline correction was performed using 5 s of prestimulus period. For EEG, the data were rereferenced according to the common average reference method. Subject-dependent band-pass filtering (3rd-order Butterworth zero-phase filter) was performed using the point-biserial correlation coefficient. The subject-dependent passbands showing the highest* r-values* were determined by a heuristic method [56]. The passbands were selected in *α*- (1 of 14 subjects), *β*- (2 of 14), *θ*- to *α*- (4 of 14), *α*- to *β*- (3 of 14), and *θ*- to *β*-bands (4 of 14).

#### 2.4.3. Classification

We performed a single trial classification of NIRS and EEG data to discriminate MA- and BL-related responses [29, 57]. To examine classification accuracy change with respect to different time windows, a sliding time window was used to extract the features of both modalities (window size: 5 s, step size: 1 s) between −5 and 25 s from stimulus onset to account for the hemodynamic delay with respect to brain activation [58]. The relatively long window size was chosen to consider the relatively slow hemodynamic responses compared to EEG, thereby increasing the performance of each modality as well as the HYB system. Both NIRS and EEG features were calculated for each sliding time window. All NIRS and EEG channels were used for feature extraction and classification (9 and 14 channels). For NIRS, the mean values and average slopes of Δ[HbO] and Δ[HbR] of each channel were calculated as NIRS features, which are widely used for NIRS data classification [7]. For EEG, the common spatial pattern (CSP) algorithm was applied to the preprocessed EEG data. EEG features were calculated as the log-scaled variance of CSP-filtered data (first and last 2 components containing the most discriminative information). The feature vectors of each sliding time window were independently used for the classification. Tenfold cross-validation was performed 10 times for each sliding window.

For classification, shrinkage linear discriminant analysis (sLDA) was used [52]. The shrinkage parameter was estimated as described previously [59, 60]. In order to confirm the advantage of adding EEG data to NIRS data, the correct answer ratio was estimated not only for the EEG or NIRS data individually but also for a combination of both modalities. For the latter case, a metaclassification approach based on sLDA was used. Normalization is not necessary when concatenating EEG and NIRS features for metaclassification. This is because, for metaclassification, both EEG and NIRS individual classifiers yield LDA-projected EEG and NIRS features with the same scale, which are combined for the input of the metaclassifier. The detailed information regarding the metaclassifier is provided in Fazli et al. [32].

## 3. Results

### 3.1. Grand Average of EEG and NIRS Data Patterns

**Figure 3:**
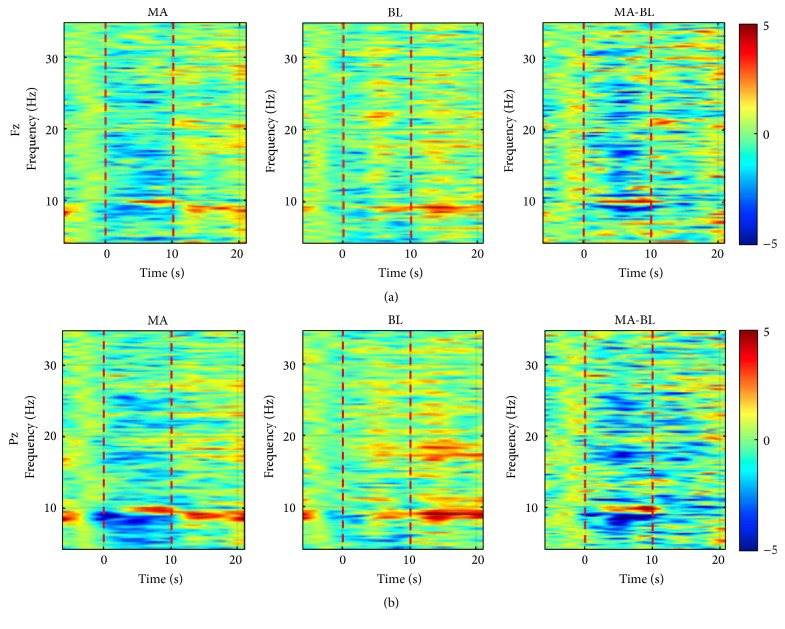
Grand average time-frequency analysis results for event-related (de)synchronization (ERD/ERS) in the frequency band of 4–35 Hz in frontal ((a) MA, BL, and MA-BL at Fz from left to right) and parietal areas ((b) MA, BL, and MA-BL at Pz from left to right).

**Figure 4:**
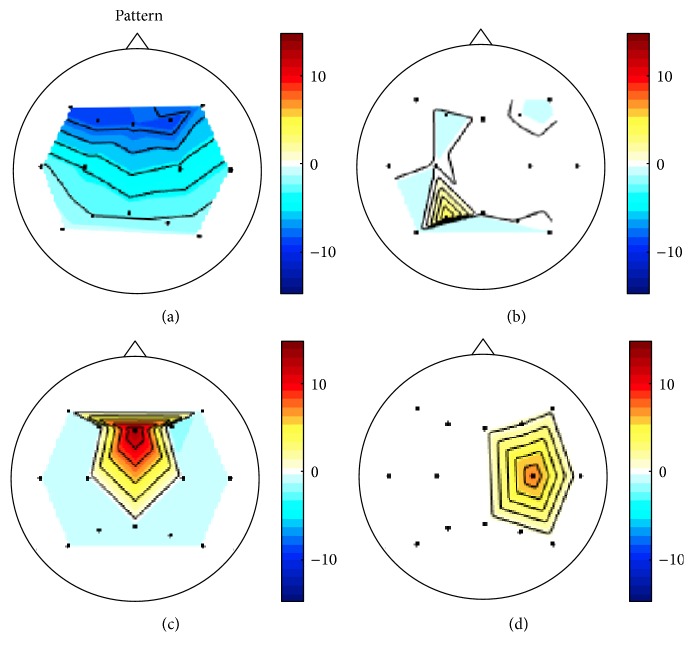
Grand average spatial patterns for all corresponding eigenvalues: *λ* = (a) 0.36, (b) 0.41, (c) 0.67, and (d) 0.77. Note that the signs of the spatial patterns are irrelevant.

**Figure 5:**
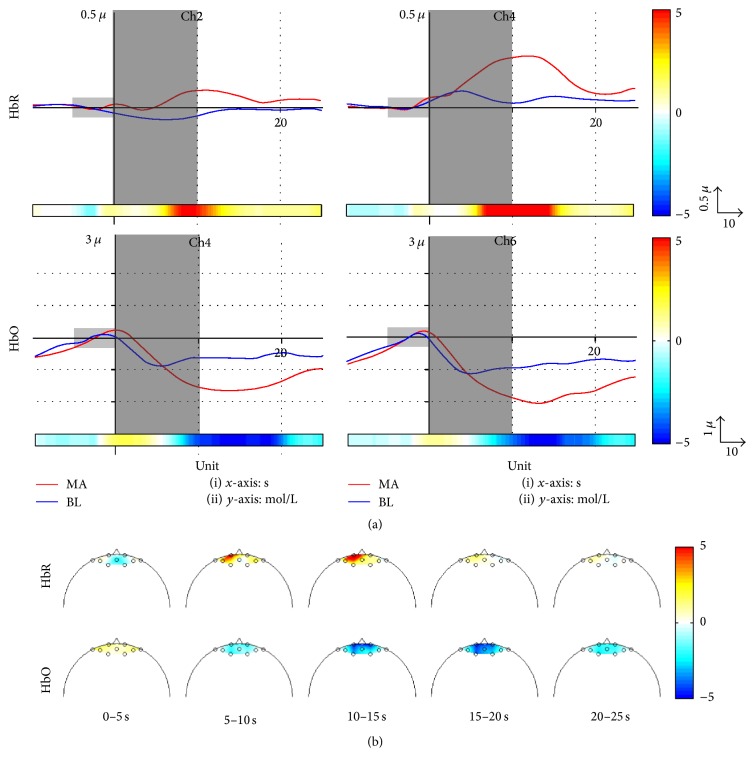
(a) Grand average time courses of changes in deoxyhemoglobin (Δ[HbR]) and oxyhemoglobin (Δ[HbO]). The log⁡(*p*) significance of each channel is shown horizontally at the bottom of each subplot. The red and blue solid lines correspond to MA-related and BL-related activation, respectively. A small gray shade depicts the baseline period of −5 to 0 s, and a large gray patch indicates the task period of 0 to 10 s. A solid vertical line indicates the onset of the task period. The units of the *x*- and *y*-axes are seconds and mol/L, respectively. (b) Time-dependent scalp plots of log⁡(*p*) significance of Δ[HbR] and Δ[HbO] based on the* r-value*. A color bar on the right side denotes a scale of log⁡(*p*) significance for both (a) and (b). The positive and negative values in the color bar indicate that MA-related activation shows higher and lower values than those of BL-related activation, respectively.

Grand average event-related (de)synchronization (ERD/ERS) patterns evoked by MA, BL, and their difference (i.e., MA-BL) at the two midline sites (Fz and Pz) in the frequency band of 4–35 Hz (theta to beta band) are shown in Figure 3. Fz and Pz represent frontal and parietal areas, respectively. Two dotted lines at* t* = 0 and 10 s denote the onset of task and rest time, respectively. During MA (0–10 s), ERDs were broadly observed ranging from *θ*- to *β*-band, while clear ERS patterns appeared in a narrow band around 10 Hz. On the other hand, fewer ERD/ERS patterns were observed during BL task. Thus, the distinct difference of ERD/ERS between MA and BL was widely observed in the corresponding frequency band. In Figure 3, *α*-band or *β*-band (8–30 Hz) is included in the passband of 12 (85.7%) or 9 (64.3%) of 14 subjects, respectively. Figures 4(a)–4(d) show the grand average of CSP patterns that correspond to the eigenvectors for the highest and lowest two eigenvalues (*λ*) for CSP [56]. Note that frontal and parietal areas are mainly associated with task-relevant activation. Figures 5(a) and 5(b) show the grand average time courses of the NIRS responses and the time-dependent scalp plot of log⁡(*p*) significance values based on the* r-value*, respectively. The red and blue solid lines in Figure 5(a) correspond to MA-related and BL-related activation, respectively, with log⁡(*p*) significance values indicated in the horizontal color bar below the curve plots. Two channels with the highest significance for each chromophore are presented, where Δ[HbO] gradually decreases and Δ[HbR] increases after onset time and they start returning to the baseline after about 15 s during MA. Compared to MA, no distinct responses are observed during BL task.  Figure 5(b) represents spatial maps of log⁡(*p*) significance values for the NIRS measurements. The color bar on the right side indicates the scale of log⁡(*p*). In the color bar, red (positive) and blue (negative) colors indicate the higher values of MA-related data and BL-related data, respectively. In the scalp plot, significant Δ[HbR] patterns on the left hemisphere are mostly due to MA, while Δ[HbO] shows a bilateral pattern. Interestingly, significant Δ[HbO] patterns appear (10–15 s) and disappear (20–25 s) with a slight delay compared with Δ[HbR].

### 3.2. Classification

Table 1 denotes the maximum accuracies of each subject among the tested time windows. Eight of eleven subjects showed the EEG accuracy exceeding the BCI performance threshold (>70% for binary communication [61]) and scored 82.0 ± 11.2% on average. All subjects exceeded the threshold accuracy when NIRS data was used (HbR + HbO) and scored 85.7 ± 4.9% on average. For all three cases combining EEG data with NIRS data, classification performance was significantly improved (e.g., HbR: 81.4 ± 7.2% versus HbR + EEG: 86.3 ± 8.4%; Wilcoxon rank-sum test, *p* < 0.05). Since HbR + HbO scored the highest mean accuracy among the tested NIRS chromophores (HbR, HbO, and HbR + HbO), HbR + HbO represents the NIRS result hereafter. The classification of HbR + HbO + EEG could enhance the accuracy by 2.5% and 6.2% compared to NIRS and EEG alone, respectively.

**Figure 6:**
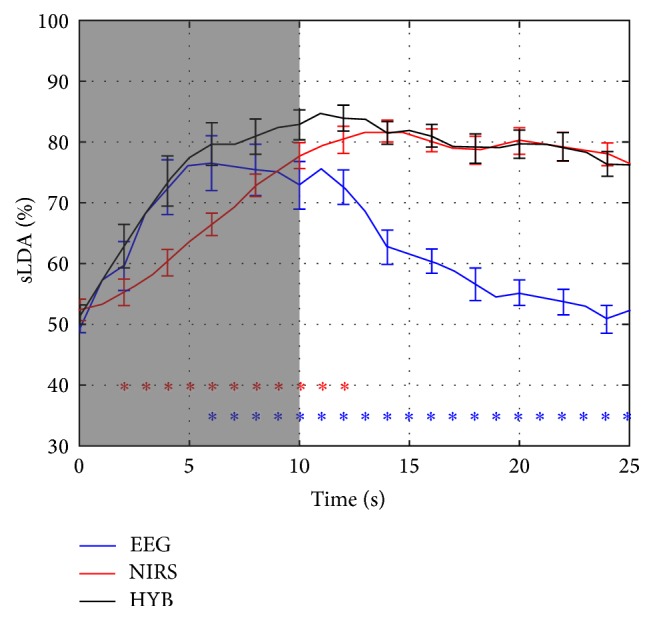
Grand average time-dependent NIRS (red), EEG (blue), and hybrid (HYB; black) classification accuracies. The gray shaded region shows a task period (*t* = 0–10 s). The red and blue asterisks below indicate the time periods in which the accuracies of HYB were significantly higher than those of NIRS (red) and EEG (blue), respectively. Error bars along with the solid lines show the standard errors.

The grand average classification accuracies with error bars indicating the standard errors are presented in Figure 6. During the task period (gray shaded period), EEG accuracy reached the highest value at* t* = 6 s. Due to the hemodynamic delay, NIRS showed the highest value 4 s after the end of the task (*t* = 14 s). The classification performance of the hybrid modality was significantly higher than that of EEG or NIRS for most time periods or was at least comparable. In Figure 6, red and blue asterisks represent time windows in which the classification accuracies of the HYB were significantly higher than those of EEG or NIRS alone, respectively.

**Figure 7:**
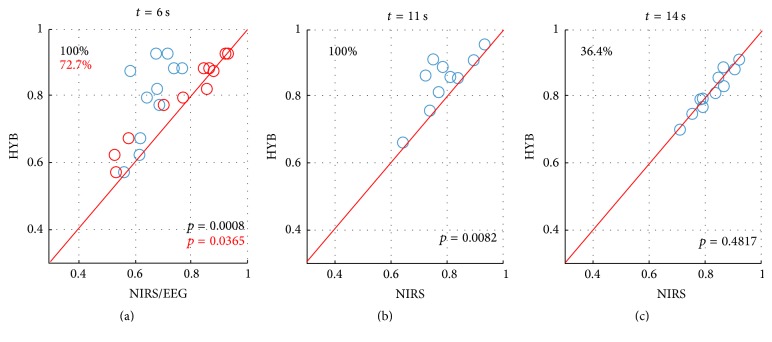
Comparisons of classification performances between NIRS and HYB (blue circles) at* t* = 6 (a), 11 (b), and 14 s (c). At* t* = 6, comparison of classification performances between EEG and HYB (red circles) and between NIRS and HYB (blue circles) is provided. The three time points are selected when EEG, hybrid, and NIRS show the highest classification accuracies according to the results shown in Figure 6. Circles above the red diagonal indicate that the performance is improved by HYB compared with NIRS/EEG. Percentage values indicate the percent of subjects showing performance improvement by HYB compared with NIRS (black) or EEG (red). *p* values indicate significance of the performance improvement by HYB compared with NIRS (black) or EEG (red).

Figure 7 shows the performance comparison between the NIRS and HYB. The comparisons were made where the EEG, HYB, and NIRS scored the maximum accuracy according to the results shown in Figure 6 (*t* = 6, 11, and 14 s, resp.). At* t* = 6, the performance comparison between the EEG and HYB was also provided (see red circles). The number on the upper left side denotes the percentage of the improved results by HYB. All subjects' performances were improved by HYB at* t* = 6 and 11 s (*p* < 0.01). At* t* = 14 s, the HYB was not capable of showing significantly better performance than NIRS (*p* = 0.482). This might be caused by less contribution of EEG features to the performance after* t* = 10 s, when less task-relevant activation was produced after the task period.

## 4. Discussion

We aimed to establish a lightweight hybrid BCI system by combining a portable NIRS with an economical EEG system. The classification results verified that the simultaneous use of EEG and NIRS data was beneficial to improve classification performance. In particular, all subjects (except one: VP001) showed increased performance when the hybrid modality was used (see Table 1). Some previous studies have already confirmed that a hybrid BCI system combining NIRS with EEG can improve system performance, but they used stationary and bulky devices, thereby limiting application outside the laboratory setting [32, 37]. Since our hybrid system was implemented by combining a portable NIRS with an economical EEG system, it can be widely used and easy to handle not only in laboratory settings but also in out-of-lab scenarios.

Even though we verified the feasibility of the hybrid neuroimaging system in a typical BCI scenario, it may also be used for neurorehabilitation purposes, such as restoring motor functions lost in neurological disorders. In this study, MA was selected as a cognitive task to demonstrate the usability of our system because it is one of the stable and consistent cognitive tasks that can produce distinct task-relevant brain activation. As the light and convenient NIRS optodes can easily be reorganized to configure the channels, they are able to measure signals from different brain areas such as motor or occipital areas. However, we must note that careful hair preparation is necessary to avoid interference with the signal acquisition in this case.

In this study, we implemented a MA-based BCI system to demonstrate the feasibility of our hybrid EEG-NIRS neuroimaging device. This device generally showed low operation speed (10 s is theoretically required for producing one command) compared to other paradigms, such as P300 and steady-state visual evoked potential (SSVEP) [62]. However, as the EEG electrodes and NIRS optodes of our hybrid device can be easily reorganized, BCI systems employing other brain areas or paradigms could also be implemented using our hybrid neuroimaging device. For example, it is possible to develop an SSVEP-based BCI system by moving the recording sensors of our hybrid system to occipital areas. It has been well documented that an SSVEP-based BCI system shows high operation speed, and, in particular, a recent study demonstrated that the simultaneous use of EEG and NIRS can further increase the operation speed of an SSVEP-based BCI system [63]. Thus, our hybrid EEG-NIRS recording device may also be used to develop a high-speed BCI system for other BCI paradigms by appropriately modifying the configuration of recording sensors.

It is generally acknowledged that increased Δ[HbO] and decreased Δ[HbR] are induced in task-relevant brain areas during performance of the corresponding task. As seen in Figure 5(a), the opposite pattern to the typical NIRS signal pattern was observed, in that the increasing trend of Δ[HbR] was synchronized with the decreasing trend of Δ[HbO] from the task onset. Note that these opposite patterns are also frequently shown in the literature [64, 65]. Particularly, significant Δ[HbO] decrease and Δ[HbR] increase were observed during MA tasks in frontal areas [64–66].

As mentioned previously, system performance improvement with respect to the classification accuracy was not accomplished by HYB after* t* = 14 s. This likely results from the lack of task-relevant EEG signals after task termination, and, therefore, EEG does not contribute to the enhancement of system performance at this time. Moreover, before* t* = 6 s, because of inferior temporal responsiveness of NIRS due to inherent hemodynamic delay, the system performance improvement is also not observed. It is worth mentioning that, because of the delayed responses of NIRS, it would be hard to implement a high-speed BCI system using solely NIRS; nevertheless, NIRS is helpful as a second modality when incorporated in a hybrid BCI with EEG. Based on changing performance over time, an optimal task time length can be determined between 6 and 12 s for HYB. However, for* t* = 6 s, the degree of performance improvement is less than that for* t* = 12 s, while 12 s may degrade the usability of the NIRS system owing to the relatively long task time. The tradeoff between the time period to make a decision and performance should be considered based on whether system speed or performance is preferred.

## 5. Conclusion

Recently, various easily wearable commercial EEG devices have been released [67–70]. These devices have lightweight and easy-to-use configurations. They are used in the field of rehabilitation as well as in entertainment. In this study, we verified the usefulness of a lightweight hybrid BCI system by combining a portable NIRS and a cost-effective EEG system. Our hybrid EEG-NIRS system allowed for improved classification performance. Despite probable doubt with respect to system stability and reliability of the economical EEG system, we verified that the proposed system is capable of stably enhancing the system performance. The concurrent use of the portable NIRS and EEG systems can help us to use the combined system more practically in a cost-effective way. Therefore the proposed system has a high potential for future BCI research in out-of-lab scenarios at low cost.

## Figures and Tables

**Table 1:** Maximum classification accuracies of each subject for near-infrared spectroscopy (NIRS), electroencephalography (EEG), and their possible combination (HYB) after onset of task period.

Subject	EEG	HbR	+EEG	HbO	+EEG	HbR + HbO	+EEG
VP001	66.8	86.3	86.0	86.7	84.7	89.0	87.5
VP002	85.8	78.2	**86.0**	85.2	84.8	84.3	**85.8**
VP003	88.7	87.2	**91.5**	87.5	**90.2**	90.5	**93.3**
VP004	67.5	82.2	82.0	72.2	**77.7**	83.0	82.5
VP005	88.3	80.8	**88.3**	83.0	**90.0**	82.0	**88.3**
VP006	96.7	90.5	**97.3**	85.8	**96.7**	93.5	**98.0**
VP007	93.8	86.8	**93.7**	89.5	**94.2**	90.3	**93.7**
VP008	88.2	81.8	**89.7**	90.5	**92.0**	89.8	**91.8**
VP009	61.5	62.3	**64.5**	74.3	**75.2**	78.3	77.0
VP010	86.8	83.2	**90.3**	76.7	**90.3**	83.3	**91.2**
VP011	78.3	76.5	**79.5**	79.5	**82.0**	78.5	**81.2**

*Mean*	82.0	81.4	86.3^**∗∗**^	82.8	87.1^**∗**^	85.7	88.2^**∗**^
*Std*	11.2	7.2	8.4	5.9	6.5	4.9	5.9

^*∗*^ *p* < 0.05 and ^*∗∗*^*p* < 0.01 (Wilcoxon signed rank-sum).

^**∗**^HbR/HbO: deoxyhemoglobin/oxyhemoglobin.

^**∗**^Std: standard deviation.
